# Supplementary material for: Evaluation of long-read sequencing for Ostreid herpesvirus type 1 genome characterization from Magallana gigas infected tissues
Source: Microbiol Spectr. 2025 Jan 23;13(3):e02082-24. doi: 10.1128/spectrum.02082-24 (PMC11878034; doi:10.1128/spectrum.02082-24)
Supplement: Supplemental figures and tables — Fig. S1, S2, and S3; Table S1. [file spectrum.02082-24-s0001.pdf]

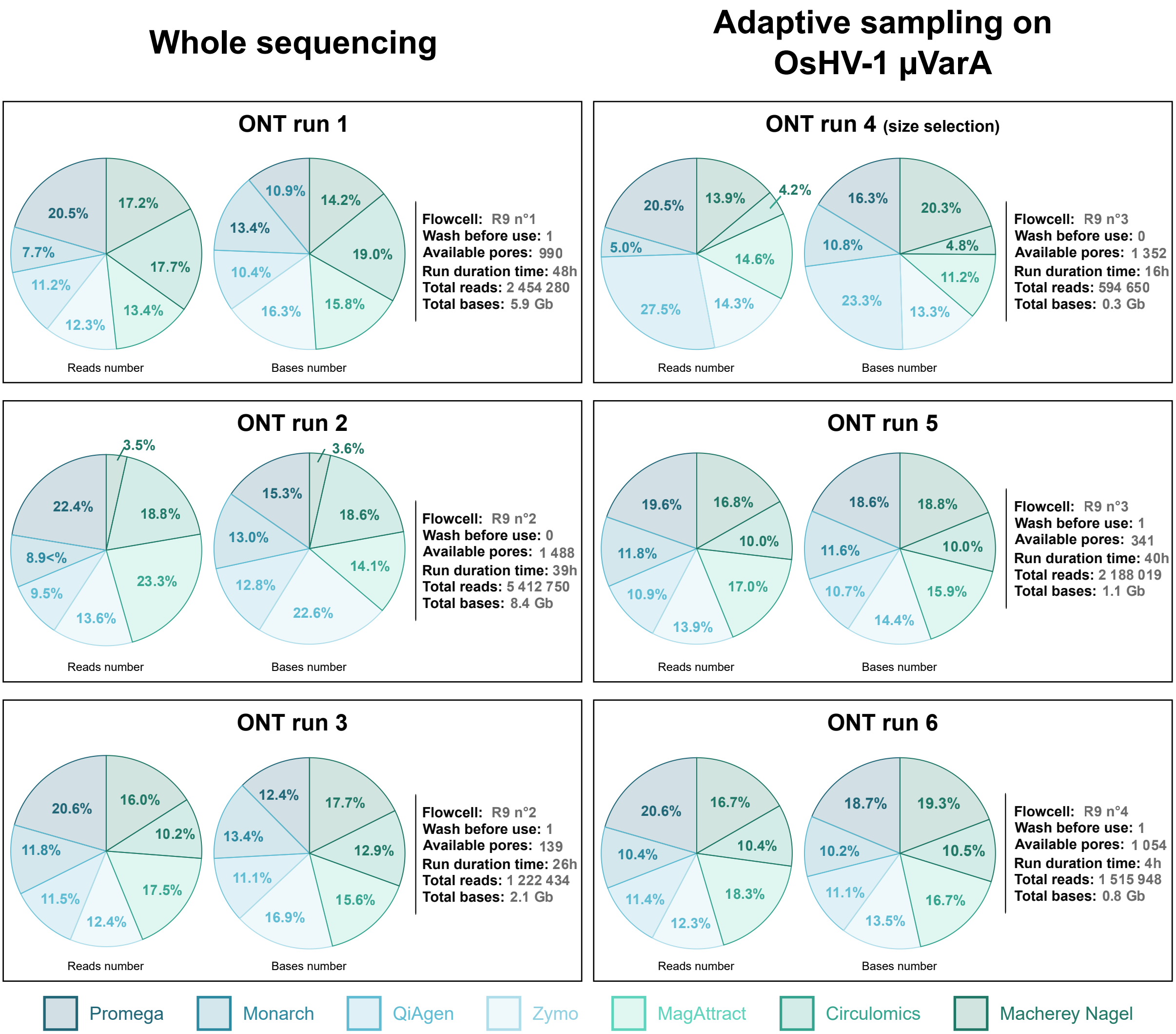

Figure Supplementary 1: Statistics of sequencing runs. Pie chart showing percentages reads sequenced and percentages of bases sequenced for each DNA samples sequenced from seven different HMW DNA extraction kits, for six ONT sequencing runs, along with some run characteristics: Flow cell batch number, number of flow cell washes, number of pores available before sequencing (i.e. flowcell check results), run duration, and total number of reads and bases sequenced.

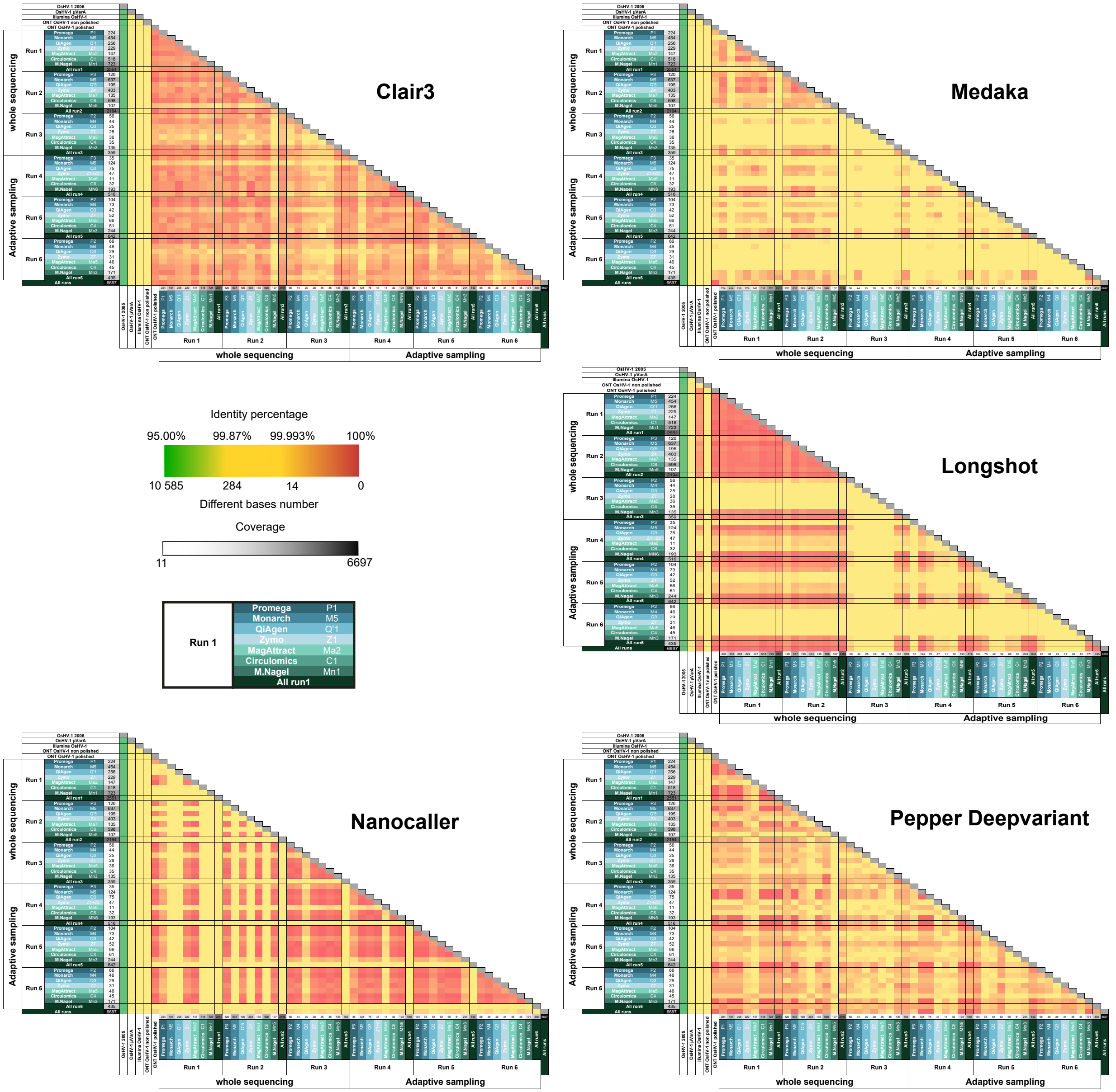

Figure Supplementary 2: Consensus caller evaluation. Each consensus caller was tested on 49 alignment files made by mapping reads coming from each sample and from each sequencing run, plus all reads pooled together from each run, plus all reads pooled together from all runs against the OsHV-1 ONT genome produced from all reads from all run and polished with medaka. The 49 OsHV-1 consensus sequences were aligned with the OsHV-1 reference sequence (GenBank: NC005881.2); OsHV-1 µVarA (GenBank: KY242785.1), Illumina NR OsHV-1, and both polished and non-polished ONT OsHV-1 genomes. Pairwise percentage of identity and base number differences are color-coded from red (100% identity) to green. Average reads coverage for each alignment is color-coded from light grey (low coverage) to dark grey (high coverage).

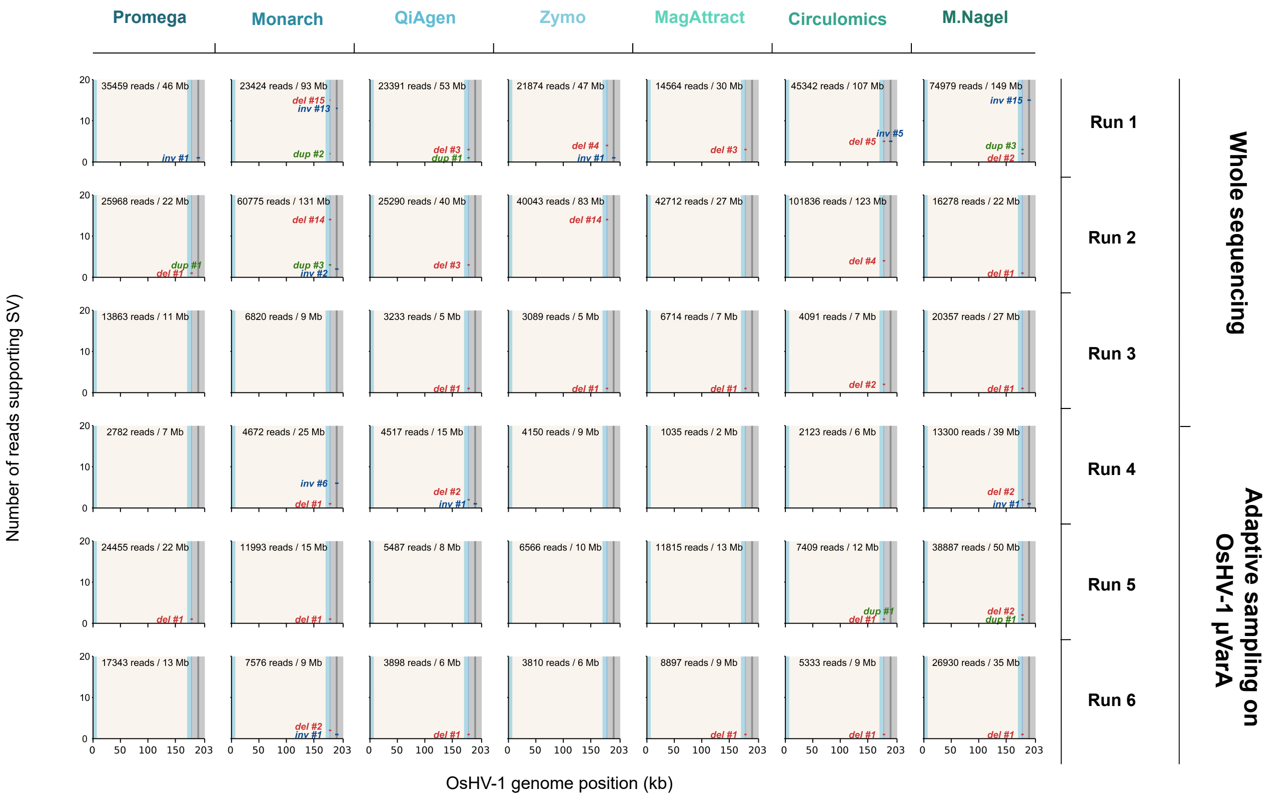

Figure Supplementary 3: Structural variation of ONT reads from each library. Identification of the major structural variations (X deletion / duplication and US inversion) detected in each of the 42 ONT libraries. The OsHV-1 structure is shown with the specific regions: TRL/IRL (turquoise); UL (white); X (blue); TRS/IRS (grey); US (dark grey).

Table Supplementary 1: Virome characterisation results

Sequence name	Seq. technology	Aligned reads nb.	Max. score	Max. score group	Length	Hallmark	Viral	Cellular	Contig Length	Total genes	Viral genes	Host genes
**contig_43**	ONT	13	**0,987**	dsDNAphage	2452	1	60	0	2506	5	4	0
**contig_695**	ONT	19	**0,507**	dsDNAphage	18293	0	14,8	3,7	18326	29	3	4
**contig_746**	ONT	177	**0,987**	lavidaviridae	6900	0	16,7	0	8467	6	1	0
**contig_883**	ONT	38	**1**	lavidaviridae	2361	0	40	0	2577	5	0	0
**contig_202**	ONT	8	**0,98**	ssDNA	1206	0	33,3	0	1291	3	0	1
**contig_885**	ONT	59	**0,98**	lavidaviridae	1897	0	33,3	0	4750	3	0	0
**contig_823**	ONT	52	**0,953**	NCLDV	3057	0	50	0	3294	4	0	1
**contig_36**	ONT	63	**0,947**	lavidaviridae	4569	0	50	0	8102	2	0	0
**contig_687**	ONT	18	**0,933**	ssDNA	2083	0	33,3	0	3758	3	0	0
**contig_435**	ONT	51	**0,9**	ssDNA	2025	0	33,3	0	3163	3	0	0
**contig_501**	ONT	68	**0,9**	NCLDV	2415	0	33,3	0	2729	3	0	0
**contig_768**	ONT	89	**0,9**	lavidaviridae	4828	0	16,7	0	5647	6	0	0
**contig_847**	ONT	5	**0,887**	dsDNAphage	3749	0	71,4	0	3751	7	0	0
**contig_1012**	ONT	319	**0,887**	NCLDV	5498	0	50	0	6236	2	0	0
**contig_347**	ONT	75	**0,88**	lavidaviridae	4527	0	50	0	4824	2	0	0
**contig_311**	ONT	71	**0,88**	lavidaviridae	2590	0	25	0	5231	5	0	0
**contig_1013**	ONT	125	**0,747**	lavidaviridae	4962	0	50	0	6963	2	0	0
**contig_1025**	ONT	344	**0,74**	ssDNA	2147	0	33,3	0	4545	3	0	0
**contig_59**	ONT	92	**0,72**	lavidaviridae	5002	0	14,3	0	5402	7	0	0
**contig_991**	ONT	652	**0,573**	lavidaviridae	10717	0	11,1	0	12055	9	0	0
**contig_905**	ONT	7	**0,5**	NCLDV	12115	0	11,1	0	14269	14	0	0
**NODE_5067**	Illumina	255	**0.953**	dsDNAphage	2505	1	40	0	2506	5	3	0
**NODE_10991**	Illumina	27	**0.987**	dsDNAphage	1744	1	50	0	1756	5	2	0
**NODE_82**	Illumina	643	**0.933**	lavidaviridae	7782	0	18,2	0	7784	11	2	0
**NODE_169**	Illumina	1693	**0.867**	lavidaviridae	6802	0	50	0	6802	6	2	0
**NODE_13874**	Illumina	80	**0.993**	ssDNA	1384	0	50	0	1549	2	1	0
**NODE_1807**	Illumina	460	**0.980**	NCLDV	2556	0	25	0	3737	6	1	0
**NODE_20410**	Illumina	17	**0.980**	lavidaviridae	1234	0	33,3	0	1237	3	1	0
**NODE_12227**	Illumina	94	**0.973**	lavidaviridae	1202	0	50	0	1657	2	1	0
**NODE_1082**	Illumina	148	**0.967**	dsDNAphage	2369	1	20	0	4327	3	1	0
**NODE_2404**	Illumina	191	**0.940**	lavidaviridae	2878	0	25	0	3393	4	1	0
**NODE_16521**	Illumina	72	**0.927**	ssDNA	989	0	50	0	1403	2	1	0
**NODE_199**	Illumina	496	**0.867**	NCLDV	6480	0	10	0	6555	13	1	1
**NODE_3364**	Illumina	177	**0.867**	ssDNA	2679	0	25	0	2984	4	1	0
**NODE_2432**	Illumina	188	**0.853**	lavidaviridae	3187	0	16,7	0	3382	6	1	0
**NODE_581**	Illumina	448	**0.820**	NCLDV	2840	0	20	0	5115	5	1	0
**NODE_3691**	Illumina	132	**0.753**	ssDNA	2872	0	25	0	2873	6	1	0
**NODE_22045**	Illumina	23	**0.687**	NCLDV	680	0	50	0	1177	1	1	0
**NODE_10532**	Illumina	205	**0.633**	lavidaviridae	1621	0	33,3	0	1795	3	1	0
**NODE_15636**	Illumina	31	**1**	lavidaviridae	1444	0	33,3	0	1446	3	0	0
**NODE_21682**	Illumina	151	**1**	ssDNA	1186	0	33,3	0	1189	3	0	0
**NODE_26231**	Illumina	30	**1**	NCLDV	992	0	50	0	1057	2	0	0
**NODE_893**	Illumina	520	**1**	lavidaviridae	4539	0	25	0	4609	4	0	0
**NODE_1208**	Illumina	270	**0,993**	lavidaviridae	4188	0	40	0	4189	5	0	0
**NODE_14558**	Illumina	54	**0,993**	ssDNA	765	0	50	0	1505	2	0	0
**NODE_25296**	Illumina	24	**0,993**	ssDNA	1082	0	33,3	0	1082	3	0	0
**NODE_6136**	Illumina	142	**0,993**	ssDNA	1104	0	50	0	2309	2	0	1
**NODE_12069**	Illumina	332	**0,987**	ssDNA	1665	0	25	0	1668	4	0	0
**NODE_15276**	Illumina	153	**0,987**	ssDNA	958	0	50	0	1464	2	0	0
**NODE_2157**	Illumina	317	**0,987**	NCLDV	2970	0	50	0	3523	1	0	0
**NODE_663**	Illumina	336	**0,987**	lavidaviridae	3325	0	33,3	0	4978	1	0	0
**NODE_3387**	Illumina	230	**0,98**	ssDNA	543	0	50	0	2976	2	0	0
**NODE_1034**	Illumina	210	**0,973**	lavidaviridae	2591	0	50	0	4399	2	0	0
**NODE_1097**	Illumina	233	**0,967**	NCLDV	3985	0	50	0	4315	2	0	0
**NODE_3726**	Illumina	132	**0,967**	NCLDV	1642	0	50	0	2862	1	0	1
**NODE_38**	Illumina	737	**0,967**	lavidaviridae	7621	0	25	0	8988	3	0	0
**NODE_496**	Illumina	367	**0,953**	lavidaviridae	3793	0	33,3	0	5345	3	0	0
**NODE_149**	Illumina	1410	**0,94**	lavidaviridae	6890	0	50	0	7086	2	0	0
**NODE_4703**	Illumina	161	**0,94**	ssDNA	1588	0	33,3	0	2588	3	0	0
**NODE_18169**	Illumina	52	**0,933**	ssDNA	1194	0	33,3	0	1328	1	0	0
**NODE_25859**	Illumina	18	**0,933**	ssDNA	1063	0	33,3	0	1067	3	0	0
**NODE_1374**	Illumina	389	**0,927**	NCLDV	4056	0	33,3	0	4057	3	0	0
**NODE_12177**	Illumina	109	**0,92**	ssDNA	833	0	50	0	1660	2	0	0
**NODE_2422**	Illumina	437	**0,913**	lavidaviridae	3154	0	25	0	3384	4	0	0
**NODE_10755**	Illumina	347	**0,907**	lavidaviridae	1703	0	50	0	1774	2	0	0
**NODE_17251**	Illumina	159	**0,907**	ssDNA	1365	0	25	0	1367	4	0	0
**NODE_26954**	Illumina	32	**0,907**	NCLDV	521	0	50	0	1037	2	0	0
**NODE_20**	Illumina	1488	**0,9**	lavidaviridae	9244	0	33,3	0	10204	4	0	0
**NODE_2154**	Illumina	185	**0,867**	lavidaviridae	3404	0	20	0	3524	6	0	0
**NODE_1748**	Illumina	594	**0,853**	ssDNA	1394	0	33,3	0	3775	3	0	0
**NODE_18962**	Illumina	26	**0,853**	ssDNA	983	0	50	0	1295	2	0	0
**NODE_1039**	Illumina	334	**0,827**	ssDNA	540	0	50	0	4392	2	0	0
**NODE_981**	Illumina	250	**0,813**	lavidaviridae	4347	0	50	0	4484	2	0	0
**NODE_304**	Illumina	1690	**0,807**	lavidaviridae	6001	0	33,3	0	6002	2	0	0
**NODE_1497**	Illumina	1497	**0,78**	ssDNA	2894	0	40	0	3955	5	0	0
**NODE_384**	Illumina	449	**0,78**	lavidaviridae	5154	0	33,3	0	5689	3	0	0
**NODE_1118**	Illumina	374	**0,767**	dsDNAphage	3981	0	12,5	0	4270	8	0	0
**NODE_160**	Illumina	505	**0,753**	lavidaviridae	6185	0	20	0	6891	5	0	0
**NODE_6729**	Illumina	105	**0,753**	NCLDV	2158	0	33,3	0	2217	1	0	0
**NODE_1134**	Illumina	268	**0,74**	lavidaviridae	1525	0	50	0	4256	2	0	0
**NODE_1478**	Illumina	252	**0,6**	NCLDV	3927	0	50	0	3969	2	0	0
**NODE_398**	Illumina	349	**0,527**	NCLDV	4243	0	50	0	5634	2	0	0
